# RETRACTION: NNK From Tobacco Smoking Enhances Pancreatic Cancer Cell Stemness and Chemoresistance by Creating a β2AR‐Akt Feedback Loop That Activates Autophagy

**DOI:** 10.1002/1878-0261.70312

**Published:** 2026-07-20

**RETRACTION**: X. Chen, W. Zhang, R. Liu, Z. Zhu, M. Gong, Q. Wang, W. Qian, Z. Wu, Q. Ma and Z. Wang, “NNK From Tobacco Smoking Enhances Pancreatic Cancer Cell Stemness and Chemoresistance by Creating a β2AR‐Akt Feedback Loop That Activates Autophagy,” *Molecular Oncology* 16, no. 15 (2022): 2881‐2895, https://doi.org/10.1002/1878‐0261.13230.

The above article, published online on 20 May 2022 in Wiley Online Library (wileyonlinelibrary.com), has been retracted by agreement between the authors, the journal Editor‐in‐Chief, Kevin Ryan; FEBS Press; and John Wiley and Sons Ltd. The retraction has been agreed following concerns raised by a third party. An investigation identified duplication between Figure 5F of this article and a figure published earlier in another article by a different author group. The authors explained that the image was used as reference material and mistakenly incorporated into the figure. The authors provided some supporting data, but this did not meet the criteria for raw data. Given the image duplication and the lack of appropriate raw data, the editors consider the results to be unreliable.

